# Effects of once-yearly zoledronic acid on bone density and incident vertebral fractures in nonmetastatic castration-sensitive prostate cancer patients with osteoporosis

**DOI:** 10.1186/s12885-021-08177-w

**Published:** 2021-04-17

**Authors:** Daisuke Watanabe, Takahiro Kimura, Ken Watanabe, Hiromitsu Takano, Yuko Uehara, Tadaaki Minowa, Akemi Yamashita, Seiichiro Yoshikawa, Akio Mizushima

**Affiliations:** 1 Department of Palliative Medicine, Juntendo University Graduate School of Medicine, 2-1-1 Hongo, Bunkyo-ku, Tokyo, 113-8421 Japan; 2 Department of Urology, Koto Hospital, Tokyo, Japan; 3 Department of Urology, The Jikei University School of Medicine, Tokyo, Japan; 4 Department of Radiology, The Jikei University School of Medicine, Tokyo, Japan; 5 Department of Orthopedic Surgery, Koto Hospital, Tokyo, Japan; 6 Cancer Therapeutic Center, Juntendo University Urayasu Hospital, Chiba, Japan

## Abstract

**Background:**

Androgen deprivation therapy (ADT) is the effective treating prostate cancer but is often accompanied by cancer treatment-induced bone loss (CTIBL), which impairs the patient’s quality of life. In patients with nonmetastatic castration-sensitive prostate cancer (M0CSPC) who already have osteoporosis before starting ADT, appropriate bone-modifying agent intervention must be performed in parallel, as the patient has a high risk of future fracture. However, little is known about therapeutic interventions aimed at preventing the progression of CTIBL and new fractures. The present study explored the effect of once-yearly zoledronic acid 5 mg (ZOL 5 mg) on bone mineral density (BMD) and new vertebral fractures (VFs) in M0CSPC patients with coexisting osteoporosis before starting ADT.

**Methods:**

We conducted a retrospective, multi-institutional, cohort study involving 42 M0CSPC patients with osteoporosis who had undergone ADT with/without a single intravenous infusion of ZOL 5 mg at the start of ADT (ZOL 5 mg group, *n* = 26; control group, *n* = 16). The association of the ZOL 5 mg with changes in the BMD from baseline to 12 months and the incidence of VFs were evaluated.

**Results:**

Prevalent VFs were found in 47.6% of all patients at baseline. ZOL 5 mg significantly increased the lumbar spine BMD (LS-BMD) (mean rate of change: + 4.02%, *p* < 0.0001) and significantly decreased the TRACP-5b (mean rate of change: − 52.1%, *p* < 0.0001) at 12 months after starting ADT. Incident VFs were identified in 19.0% of all patients at 12 months after starting ADT. After adjusting for the age, BMI, and changes in the LS-BMD, ZOL 5 mg was not significantly associated with incident VFs (odds ratio 0.66, 95% confidence interval 0.04–11.3, *p* = 0.7774).

**Conclusion:**

ZOL 5 mg significantly increased the LS-BMD 12 months after starting ADT, and our short-term results showed that ZOL 5 mg was not significantly correlated with the suppression of incident vertebral fractures.

## Background

Androgen deprivation therapy (ADT), which forms the core of prostate cancer treatment, reduces the overall bone strength by decreasing the bone mineral density (BMD) and consequently the bone quality [1, 2]. Osteopenia and osteoporosis associated with such cancer treatment are defined as cancer treatment-induced bone loss (CTIBL) [3]. CTIBL induces fractures and can be an important adverse event that not only reduces patients’ quality of life but also shortens the overall survival [4–6]. In a large cohort study of more than 20,000 people in New Zealand, ADT application was associated with a 2.83-fold increase in “a fracture risk requiring hospitalization” adjusted for age and ethnicity [7]. Patients undergoing ADT are presumed to have a fracture risk equal to or greater than that of patients with primary osteoporosis and existing fractures [3, 7]. If patients scheduled to undergo ADT already have osteoporosis, appropriate bone-modifying agent (BMA) interventions should be performed in parallel, as these patients are at an increased risk of fracture [3].

The Japanese management manual for CTIBL caused by ADT does not specify a first-line drug for the treatment of osteoporosis, only suggesting good treatment outcomes for zoledronic acid (ZOL) and denosumab [3]. Little is known about therapeutic interventions aimed at stopping the progression of CTIBL and preventing fractures in patients with nonmetastatic castration-sensitive prostate cancer (M0CSPC) who already have osteoporosis before starting ADT. To date, the only drug that has been proven useful for fracture prevention is denosumab 60 mg administered once every 6 months, as reported in a large-scale randomized control trial (RCT) [8]. Few studies have examined the utility of other osteoporosis treatments, including annual intravenous administration of ZOL 5 mg, which was approved in Japan in 2016. To our knowledge, no studies have investigated the effect of once-yearly ZOL 5 mg on the BMD or development of new vertebral fractures in patients with M0CSPC who already had osteoporosis before starting ADT.

Given the above, the present study clarified the effects of once-yearly ZOL 5 mg on BMD and the development of new vertebral fractures in Japanese men with M0CSPC who had osteoporosis before starting ADT.

## Methods

### Patients

In this retrospective multi-institutional cohort study, from January 2016 to December 2017, we retrospectively evaluated 108 patients who were diagnosed with prostate cancer and were newly started on ADT for ≥12 months at Koto Hospital (Tokyo, Japan) and Jikei University Hospital (Minato-ku, Tokyo, Japan). The inclusion criteria were that the patient had osteoporosis that required treatment prior to starting ADT, with the following definition: 1) there was a proximal femoral or vertebral fracture, and/or 2) the BMD T score (femoral neck or lumbar spine) before starting ADT was ≤ − 2.5 [9]. The exclusion criteria were as follows: 1) cases with bone metastasis before starting ADT, 2) cases with dual energy X-ray absorptiometry (DXA) images, X-ray/CT images and blood test results could not be confirmed at the start of ADT and 12 months later, and 3) cases in which a BMA other than ZOL 5 mg was administered during the 12-month treatment period of ADT. The final study included 42 patients, divided into two groups: an osteoporosis group that received ZOL 5 mg at the start of ADT (ZOL 5 mg group, *n* = 26) and an osteoporosis group that did not receive ZOL 5 mg (control group, *n* = 16).

At the time this study was conducted, all patients diagnosed with osteoporosis based on the latest DXA images underwent BMA intervention or orthopedic consultation by the attending physician.

The study’s retrospective protocol was conducted according to the guidelines of the Declaration of Helsinki and approved by the Ethics Committee of Koto Hospital (no. 202062, approved on Jun. 15, 2020) and The Jikei University School of Medicine (32–123(10199), approved on Sep. 29, 2020).

### Evaluation

The primary outcome evaluated in this study was the occurrence of incident vertebral fracture. A spine surgeon (M.T.) and a radiologist (K.W.) examined the chest-to-pelvis sagittal images of CT or X-ray photographs performed before and 12 months after the start of ADT. The anterior, central and posterior heights of each vertebral body from Th1 to L5 were measured. Vertebral fracture was diagnosed if at least 1 of these 3 measurements was reduced by more than 20% compared to the height of the nearest uncompressed vertebral body [10]. Vertebral fractures were classified into prevalent vertebral fractures and incident vertebral fractures on vertebral body images before and 12 months after the start of ADT. The BMD was evaluated at the lumbar spine (L1–4) and femoral neck by DXA using Lunar Prodigy (GE Lunar Corp., Madison, WI, USA).

Furthermore, clinical data, including the age, body height, weight, body mass index (BMI), serum creatinine levels, estimated glomerular filtration rate (eGFR), tartrate-resistant acid phosphatase 5b (TRACP-5b), prostate-specific antigen (PSA), and medical history (diabetes mellitus, hypertension, dyslipidemia), were also collected. ZOL 5 mg is a renal excretion-type drug, and proximal tubular disorders and acute renal failure have been reported as side effects of unknown frequency [11]. The mechanism of action of ZOL on main bone involves the induction of osteoclast apoptosis and the inhibition of bone resorption due to a function loss. TRACP-5b is an enzyme that exists only in osteoclasts, and its blood concentration increases as bone resorption increases [12]. In this study, the values of renal function markers (creatinine levels, eGFR) and bone metabolism markers (TRACP-5b) measured before and 12 months after the start of ADT were investigated.

### Statistical analysis

Differences in clinical parameters between the ZOL 5 mg group and the control group were determined by Student’s *t*-test or the chi-square test. The paired *t*-test was used to compare continuous variables between 2 paired groups before and after 12 months of ADT. A logistic regression analysis was used to assess the effect of ZOL 5 mg on incident vertebral fractures. *P* values of < 0.05 were considered to indicate statistical significance. All statistical analyses were performed using the JMP® 14 software program (SAS Institute Inc., Cary, NC, USA).

## Results

### Patient characteristics

The baseline characteristics of 42 patients with M0CSPC who had osteoporosis are shown in Table 1. Ten of the 26 patients (38.5%) in the ZOL 5 mg group and 10 of the 16 patients (62.5%) in the control group were diagnosed with a prevalent vertebral fracture, showing no significant difference between the groups. In addition, no significant differences were observed between the groups in the baseline age, body height, weight, BMI, serum creatinine levels, eGFR, TRACP-5b, PSA, lumbar spine BMD (LS-BMD), femoral neck BMD (FN-BMD) or prevalence of diabetes mellitus, hypertension or dyslipidemia. In the present study, the type of ADT in all patients was combined androgen blockade using both luteinizing hormone-releasing hormone agonists and bicalutamide, with a duration of more than 12 months. No hip fractures were noted during the 12 months of ADT in either group in this study. Adverse events were observed in 2 (7.7%) of the 26 patients who received ZOL 5 mg, namely a fever and influenza-like symptoms in 1 and tetany due to hypocalcemia in the other.

**Table 1:** Baseline characteristics of M0CSPC patients with osteoporosis

	ZOL 5 mg group (***n*** = 26)	Control group (***n*** = 16)	***p***
Median age, years (range)	75 (63–85)	77 (70–89)	0.1884
Mean body height, m (SD; range)	1.63 (0.08; 1.41–1.72)	1.63 (0.06; 1.52–1.73)	0.8598
Mean weight, kg (SD; range)	61.9 (9.65; 43.8–80.0)	56.9 (10.9; 41.6–80.0)	0.1326
Mean BMI, kg/m^2^ (SD; range)	23.3 (2.89; 15.7–27.5)	21.3 (3.65; 15.4–30.1)	0.0603
Mean Creatinine, mg/dl (SD; range)	0.84 (0.16; 0.57–1.2)	0.92 (0.26; 0.64–1.5)	0.2377
Mean eGFR, ml/min/1.73 m^2^ (SD; range)	71.4 (16.0; 45.2–104.7)	65.9 (16.4; 34.8–89.2)	0.2893
Mean TRACP-5b, mU/dl (SD; range)	560.5 (198.6; 268–994)	457.9 (227.9; 178–1180)	0.1758
Mean PSA, ng/ml (SD; range)	18.8 (16.2; 4.55–58.59)	33.2 (48.3; 5.77–205)	0.1683
Diabetes mellitus, n (%)	4 (15.4)	4 (25.0)	0.4459
Hypertension, n (%)	15 (57.7)	8 (50.0)	0.6269
Dyslipidemia, n (%)	10 (38.5)	5 (31.3)	0.6343
Mean BMD, T-score (SD; range)			
Lumbar spine	−0.05 (1.67; − 1.9–4.6)	−0.86 (1.52; − 3.7–2.1)	0.1223
Femoral neck	− 2.22 (0.83; − 3.5–-0.2)	−2.16 (0.60; − 3.2–-1.2)	0.8139
Prevalent vertebral fracture, n (%)	10 (38.5)	10 (62.5)	0.1283

Differences between the groups were determined by Students’ t-test or the chi-squared test

*BMD* bone mineral density, *BMI* body mass index, *M0CSPC* nonmetastatic castration-sensitive prostate cancer, *PSA* prostate specific antigen, *SD* standard deviation, *TRACP-5b* tartrate-resistant acid phosphatase 5b, *ZOL* zoledronic acid

### BMD changes

Table 2 shows the changes in the BMD, TRACP-5b, and renal function markers due to ADT over 12 months in the ZOL 5 mg group and control group. In the ZOL 5 mg group, the LS-BMD (1.11 ± 0.25 g/cm^2^) at 12 months after ADT was significantly higher than the baseline value (1.06 ± 0.21 g/cm^2^) (*p* < 0.0001), and no significant change in the FN-BMD was observed. The average rate of change in the LS-BMD with ZOL 5 mg in combination with ADT was + 4.02%. In the control group, the LS-BMD (1.03 ± 0.16 g/cm^2^) and FN-BMD (0.76 ± 0.09 g/cm^2^) after 12 months of ADT were significantly lower (*p* = 0.0018 and *p* = 0.01, respectively) than the baseline values (1.06 ± 0.16 g/cm^2^, 0.78 ± 0.11 g/cm^2^, respectively). On comparing the rate of change between the two groups, the percentage changes in the LS-BMD and FN-BMD of the ZOL 5 mg group were significantly higher than those of the control group (*p* < 0.0001 and *p* = 0.0399, respectively).

**Table 2:** Changes in clinical parameters from baseline to 12 months after ADT for M0CSPC patients with osteoporosis

	ZOL 5 mg group (***n*** = 26)			Control group (***n*** = 16)		
	Baseline	12 months	% Change	Baseline	12 months	% Change
BMD (g/cm^2^)						
Lumbar spine	1.06 ± 0.21	1.11 ± 0.25	+ 4.02 ± 3.61 ^d, f^	1.06 ± 0.16	1.03 ± 0.16	−3.72 ± 3.91 ^b^
Femoral neck	0.73 ± 0.09	0.73 ± 0.10	+ 0.99 ± 4.41 ^e^	0.78 ± 0.11	0.76 ± 0.09	−1.52 ± 2.14 ^a^
Serum biochemistry						
Creatinine (mg/dl)	0.84 ± 0.16	0.81 ± 0.15	− 2.65 ± 11.1	0.99 ± 0.27	0.97 ± 0.24	+ 1.22 ± 10.0
eGFR (ml/min/1.73 m^2^)	71.4 ± 16.0	73.5 ± 14.9	+ 4.21 ± 13.9	61.5 ± 15.3	61.5 ± 13.4	−0.57 ± 10.8
TRACP-5b (mU/dl)	560.5 ± 198.6	247.2 ± 75.5	−52.1 ± 19.0 ^d, f^	390.9 ± 227.9	545.4 ± 286.8	+ 36.4 ± 34.9 ^c^

Data are shown as the means ± SD

*BMD* bone mineral density, *M0CSPC* nonmetastatic castration-sensitive prostate cancer, *SD* standard deviation, *TRACP-5b* tartrate-resistant acid phosphatase 5b, *ZOL* zoledronic acid

^a^ *p* < 0.05, ^b^ *p* < 0.01, ^c^ *p* < 0.001, ^d^ *p* < 0.0001 compared with baseline by paired *t*-test

^e^ *p* < 0.05, ^f^ *p* < 0.0001 compared with % change of control group by Students’ *t*-test

### Changes in TRACP-5b and renal function markers

Regarding TRACP-5b, a significant decrease compared with the baseline (mean rate of change: − 52.1%, *p* < 0.0001) was observed in the ZOL5 mg group, while a significant increase (mean rate of change: + 36.4%, *p* = 0.0003) was observed in the control group. No significant changes were observed in the serum creatinine levels or eGFR in either group. On comparing the change rates of each marker between the two groups, the ZOL 5 mg group had a significantly lower percentage change in TRACP-5b than the control group (*p* < 0.0001). The serum creatinine levels and percentage change in the eGFR were not significantly different between the two groups.

### Effects of ZOL 5 mg on incident vertebral fracture

Incident vertebral fractures were identified in 8 of 42 patients at 12 months after the start of ADT (ZOL 5 mg group: 6 patients, control group: 2 patients). The effect of ZOL 5 mg in combination with ADT on incident vertebral fractures was evaluated by a logistic regression analysis (Table 3). The logistic regression analysis with adjusting for the age, BMI, and percentage change in LS-BMD showed that the administration of ZOL 5 mg was not significantly associated with the occurrence of incident vertebral fractures (odds ratio [OR] 0.66, 95% confidence interval [CI] 0.04–11.3, *p* = 0.7774).

**Table 3:** Association between ZOL 5 mg and presence of incident VFs evaluated by a logistic regression analysis

Parameter	Presence of incident VFs		
	OR	95%CI	***p***
Administration of ZOL 5 mg	2.10	0.37–11.9	0.4033
Adjusted for age	2.19	0.37–12.9	0.3683
Adjusted for age, BMI	4.49	0.56–36.2	0.1279
Adjusted for age, BMI, ∆ LS-BMD	0.66	0.04–11.3	0.7774

*BMD* bone mineral density, *BMI* body mass index, *CI* confidence interval, *LS* lumbar spine, *VFs* vertebral fractures, *∆* percent changes

## Discussion

In this study, once-yearly ZOL 5 mg significantly increased the LS-BMD in M0CSPC patients with osteoporosis who were introduced to ADT for the first time and significantly decreased the levels of the bone resorption marker TRACP-5b. The mean rate of change in the LS-BMD for 12 months with ZOL 5 mg in combination with ADT was + 4.02% from baseline, a result that was nearly consistent with the results of an RCT performed in general men with osteoporosis treated with once-yearly ZOL 5 mg [13]. In contrast, in osteoporotic M0CSPC patients without BMA intervention, 12-month ADT significantly reduced both the LS-BMD and FN-BMD (mean rate of change: − 3.72, − 1.52%, respectively). The administration of ZOL 5 mg was not significantly associated with the suppression of incident vertebral fractures during the first 12 months after starting ADT.

Under present circumstances, urologists may not be very concerned about osteoporosis. In fact, it has been reported that too few ADT-introduced prostate cancer patients are being screened for BMD and that many patients in need of treatment for osteoporosis remain untreated [1, 14]. When diagnosing osteoporosis, not only a BMD screening but also an evaluation for existing fractures (proximal femur fractures and vertebral fractures) are important factors. Existing fractures increase the risk of future fractures by 1.86- to 2.0-fold, regardless of gender [15, 16]. In particular, prevalent vertebral fractures are strong predictors of future vertebral fractures, and a pooled analysis of published studies reported that the risk of future incident vertebral fractures increased by 4.4 times with prevalent vertebral fractures [16]. At present, the prevalence of osteoporosis in patients with non-metastatic prostate cancer before starting ADT has been reported to be 6.3 to 12.6% in Japan [17, 18]. Furthermore, the prevalence of prevalent vertebral fractures before starting ADT is estimated to be 18.8 to 21.6% [17, 19]. Although there are differences in individual study designs and the number of samples, these reports may suggest a discrepancy between the number of reported M0CSPC patients with osteoporosis and the number of osteoporosis patients who actually need treatment. Twelve months after the start of ADT, our study identified an incident vertebral fracture in 8 of 42 prostate cancer patients who had osteoporosis in need of treatment before starting ADT. Interestingly, 5 of these 8 patients (62%) with an incident vertebral fracture had already had a vertebral fracture before starting ADT. At the time of the prostate cancer diagnosis, it is considered meaningful to identify prevalent vertebral fracture by morphologically evaluating the spine using CT, which was performed in almost all of the present study’s patients for the purpose of staging, in order to avoid underdiagnosing osteoporosis. At the same time, this approach may play a complementary role in cases where urologists have not performed an initial BMD screening prior to starting ADT, thus increasing the rate of osteoporosis intervention in cases where fractures can be prevented.

The CTIBL management manual in Japan does not specify a first-line osteoporosis drug for cancer patients undergoing hormone depletion treatment, only suggesting good treatment results with zoledronic acid and denosumab [3]. There have been several studies concerning BMA interventions aimed at preventing new fractures in patients with M0CSPC in combination with ADT. A large-scale RCT provided evidence that denosumab 60 mg by subcutaneous injection every 6 months prevents incident vertebral fracture in patients with M0CSPC [8]. Although there have been differences in the observation period (from 12 to 48 months) among studies, several previously reported RCTs of ZOL 4 mg once every 3 weeks in patients with M0CSPC all showed an increase in the LS-BMD, with none reporting a preventive effect on incident vertebral fracture [20–25]. In a recently reported 2-year RCT, in which a single dose of ZOL 5 mg was administered to M0CSPC patients who were started on ADT for the first time, it was shown that the significant therapeutic effect on the areal BMD of the lumbar spine and the entire femur, although the preventive effect on new fractures was not mentioned [2]. In addition, not all patients in these studies were diagnosed with osteoporosis at baseline. In these RCTs using ZOL with different dosing intervals and durations, the BMD was a robust known surrogate marker of non-traumatic fracture risk in men, suggesting that ZOL may contribute to fracture reduction by preventing a long-term decline in the BMD [21, 25]. In our study, the administration of once-yearly ZOL 5 mg significantly increased the LS-BMD at 12 months after starting ADT in M0CSPC patients with osteoporosis but was not significantly associated with the suppression of incident vertebral fracture. This result is partially consistent with the findings of a previously reported RCT concerning the administration of once-yearly ZOL 4 mg to M0CSPC with a BMD T score > − 2.5 [26]. The lack of an association between a significant improvement in the BMD and a reduction in incident vertebral fracture in our study may be due to the lack of the tabulation of important fracture risk factors at baseline and the short observation period. Known predictors of fracture in older men, such as quadriceps strength, postural stability, a family history of fracture, alcohol intake, current smoking history, and baseline vitamin D sufficiency, were not controlled in our cohort [23, 25]. In addition, CTIBL-induced fractures increase with increasing duration of ADT treatment [27], and fracture incidence might be low during the 1-year observation period, so there is no power in our study to examine the fracture reduction associated with ZOL 5 mg. Although we did not find a significant difference, in a multivariate analysis including change in the BMD, a known surrogate marker of fracture risk in elderly men [28], there was a trend toward a reduction in incident VFs with ZOL 5 mg (OR 0.66, *p* = 0.7774), suggesting the potential efficacy of ZOL 5 mg for bone management in M0CSPC patients with osteoporosis. Further studies on the association between once-yearly ZOL 5 mg administration to M0CSPC patients with osteoporosis and the risk of new fractures are required.

In general, one of the issues with drug therapy in osteoporosis patients is the need to improve the treatment continuation rate. A cohort study of 40,002 people treated for osteoporosis in Pennsylvania, USA, reported that 45.2% were not taking the drug as prescribed at 1 year after the start of treatment, and 52.1% had dropped out by 5 years later [29]. As in other countries, Japan is similarly plagued by the issue of the therapeutic effect not being maintained due to a reduction in the treatment continuation rate [30]. In a cohort study of 12,230 Japanese osteoporosis patients that examined medication compliance and retention rates for daily, weekly, and monthly administration methods of bisphosphonates over a 5-year period, it was suggested that the longer the dosing interval, the higher the treatment continuation rate, and improvement in medication adherence could consequently be expected [30]. Most prostate cancer patients are elderly men, and they often have polypharmacy because they are taking medications for multiple comorbidities in addition to ADT (nonsteroidal antiandrogens, gonadotropin-releasing hormone analogues, etc.) for prostate cancer. Prostate cancer is a relatively slow-growing lesion compared to other carcinomas, and patients with non-metastatic locally advanced prostate cancer often have a longer duration of ADT than others [31]. If evidence of a long-term fracture-preventing effect of ZOL 5 mg in M0CSPC patients with osteoporosis were to be revealed in the future, the administration of this less burdensome annual dose might help increase the treatment continuation rate.

Several limitations associated with the present study warrant mention. First, this was a retrospective observational study, and the assessed sample size and 12-month observation period were not sufficient for evaluating the association between ZOL 5 mg and fracture risks. In this short observation period, there were not many cases in which incident vertebral fractures were confirmed. To validate the new fracture suppression effect, an RCT in which once-yearly ZOL 5 mg is administered for a longer period of time with a continuous evaluation should be conducted. Second, all M0CSPC patients included in this study were Japanese. The plasma levels of insulin-like growth factor-I (IGF-I) and IGF-binding protein-3, daily physical activity and the BMI, which can affect bone metabolism and fracture development, have been reported to differ among races [32–34]. The results of the present study may therefore not be applicable to races other than Japanese.

## Conclusions

In summary, once-yearly ZOL 5 mg significantly increased the LS-BMD at 12 months in M0CSPC patients with osteoporosis who were introduced to ADT for the first time and significantly reduced the level of TRACP-5b, a bone resorption marker. The results of this study, only 12 months in duration, showed that ZOL 5 mg did not correlate significantly with the suppression of incident vertebral fractures. Further studies are needed to elucidate the correlation between ZOL 5 mg and the risk of incident vertebral fractures with long-term follow-up.

## Data Availability

The datasets used and/or analysed during the current study available from the corresponding author on reasonable request.

## Abbreviations

ADT: Androgen deprivation therapy
BMA: Bone-modifying agent
BMD: Bone mineral density
BMI: Body mass index
CTIBL: Cancer treatment-induced bone loss
DXA: Dual energy X-ray absorptiometry
eGFR: Estimated glomerular filtration rate
FN-BMD: Femoral neck BMD
IGF: Insulin-like growth factor
LS-BMD: Lumbar spine BMD
M0CSPC: Nonmetastatic castration-sensitive prostate cancer
PSA: Prostate-specific antigen
RCT: Randomized control trial
TRACP-5b: Tartrate-resistant acid phosphatase 5b
ZOL: Zoledronic acid
